# What are the core concerns of policy analysis? A multidisciplinary investigation based on in-depth bibliometric analysis

**DOI:** 10.1057/s41599-023-01703-0

**Published:** 2023-05-01

**Authors:** Yuxue Yang, Xuejiao Tan, Yafei Shi, Jun Deng

**Affiliations:** 1 Army Medical University, Chongqing, China; 2 General Hospital of Xinjiang Military Command, Urumqi, China

**Keywords:** Social policy, Medical humanities, Environmental studies

## Abstract

Policy analysis provides multiple methods and tools for generating and transforming policy-relevant information and supporting policy evolution to address emerging social problems. In this study, a bibliometric analysis of a large number of studies on historical policy analysis was performed to provide a comprehensive understanding of the distribution and evolution of policy problems in different fields among countries. The analysis indicates that policy analysis has been a great concern for scholars in recent two decades, and is involved in multiple disciplines, among which the dominant ones are medicine, environment, energy and economy. The major concerns of policy analysts and scholars are human health needs, environmental pressures, energy consumption caused by economic growth and urbanization, and the resulting demand for sustainable development. The multidisciplinary dialog implies the complicated real-world social problems that calls for more endeavors to develop a harmonious society. A global profiling for policy analysis demonstrates that the central policy problems and the corresponding options align with national development, for example, developing countries represented by China are faced with greater environmental pressures after experiencing extensive economic growth, while developed countries such as the USA and the UK pay more attention to the social issues of health and economic transformation. Exploring the differences in policy priorities among countries can provide a new inspiration for further dialog and cooperation on the development of the international community in the future.

## Introduction

Social problems are evolving with the rapid development of economy, and the problems mankind is facing and options they choose reflect the developmental demand. Policy is a political action with specific subjects, targets, and strategies in a certain period of time, which primarily aims to create a healthy environment for the development of society (Porter, 1998; Lasswell and Kaplan, 1950; Yang et al., 2020). As for policy analysis, the definition varies a lot. According to William Dunn (2015), policy analysis is ‘an applied social science discipline, which uses multiple methods of inquiry and argument to produce and transform policy-relevant information that may be utilized in political settings to resolve policy problems.’ Jabal et al. (2019) defined that policy analysis provides methods and tools for assessing whether a policy is ‘correct and fit for their use’ and supporting policy evolution. Manski (2019) regarded policy analysis as a shorthand term that describes the process of scientific evaluation for the impact of past public policies and prediction of the potential outcomes of future policies. More generically, policy analysis is aimed to understand who develops and implements certain policies, for whom, by what, with what effects, and what techniques and tools can be used, and so on (Blackmore and Lauder, 2005; Collins, 2005).

**Fig. 1:**
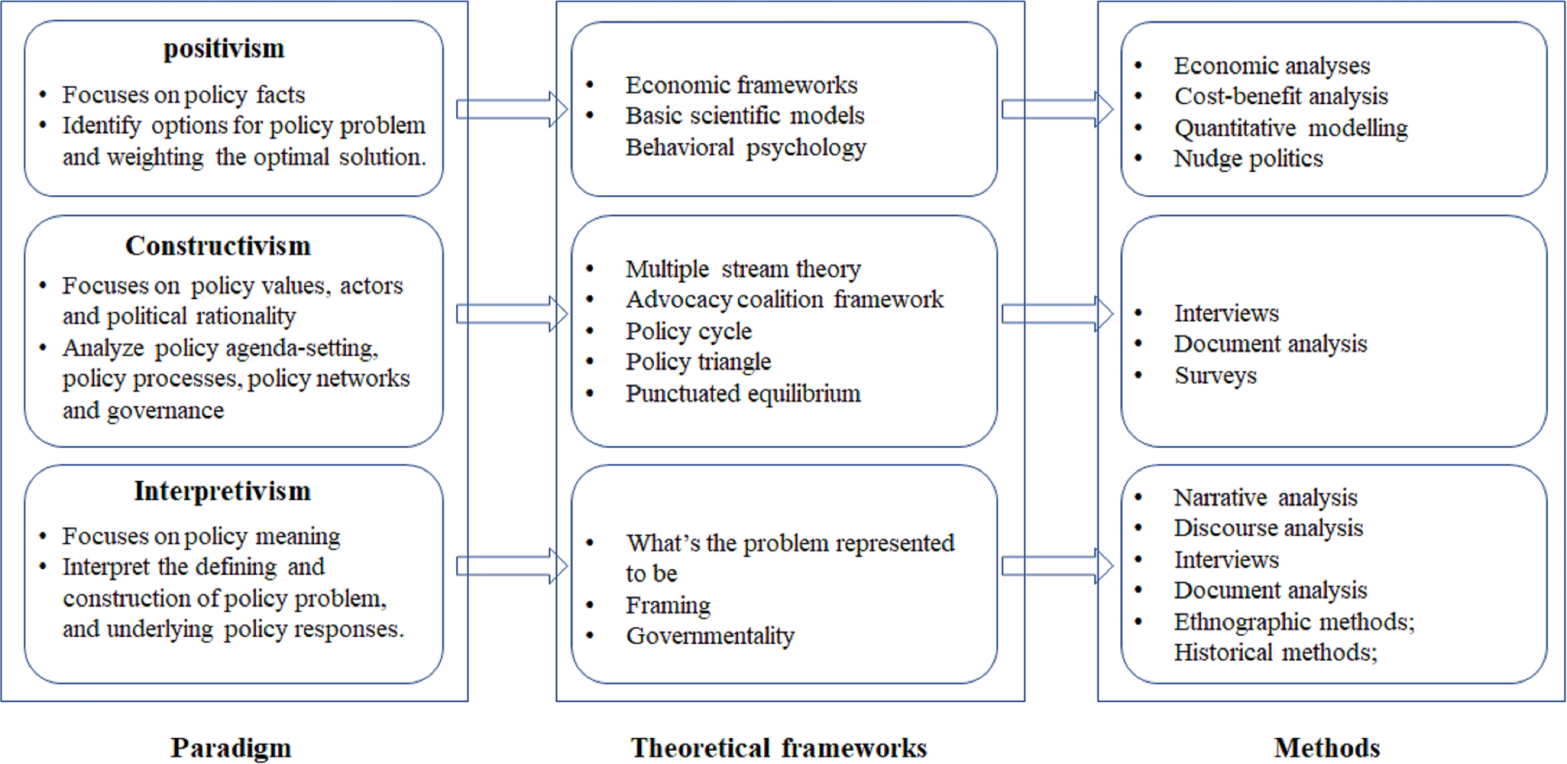
The typology of policy analysis. The framework was organized according to Jennifer et al. (2018).

Accordingly, regarding the typology of policy analysis, three categories can be established based on ontology and epistemology (Fig. 1) (Bacchi, 1999; Colebatch, 2006; Jennifer et al., 2018): (1) Positivism paradigm. Focusing on policy facts, this orientation of policy analysis aims to identify policy problems and weighting the optimal solution guided by the theory of economic frameworks, basic scientific models, and behavioral psychology through objective analysis. Economic analysis, cost-benefit analysis, quantitative modeling and nudge politics are the most commonly used methods in this orientation (Althaus et al., 2013; Jennifer et al., 2018); (2) Constructivism paradigm. In this orientation, policy is conceptualized as ‘the interaction of values, interests and resources guided through institutions and mediated through politics’ (Davis et al., 1993) rather than a comprehensively rational and linear process in which analysis involves policy agenda setting, policy processes, policy networks and governance, mainly focusing on values, actors and political rationality of policy. Theoretical frameworks, such as multiple stream theory, behavioral psychology and advocacy coalition framework, etc. are typically used in such orientation (Kingdon, 1984; Browne et al., 2019; Sabatier and Weible, 2014); (3) Interpretivism paradigm. This orientation is focused on interpreting how policy problems can be defined or constructed and how the problem framing shapes the possible policy responses (Bardach, 2000). A substantial body of research has discussed the theory underlying the problem, framing and governmentality using narrative analysis, discourse analysis, ethnographic methods, etc. (Hajer, 1995; Hajer, 2006; Martson and Mcdonald, 2006). Therefore, a systematic review of policy analysis can present the past and present policy problems of concern and the relevant possible options from an evolutionary perspective.

The profoundly complex and diversified realistic demands such as equity and sustainability (Akadiri et al., 2020), the changes of energy planning (Banerjee et al., 2000; Pandey et al., 2000; Pandey, 2002) and transition of modern markets (Blackman and Wu, 1999) have important implication on policy decisions (Munda, 2004). A multidisciplinary investigation on policy analysis can provide more reflections on how to develop a harmonious society. Studies have shown that the priority of policy agenda is determined by three key factors: the nature of the issue (Shiffman and Smith, 2007), the policy environment (Adams and Judd, 2016; Sweileh, 2021) and the capabilities of proponents (Shawar and Shiffman, 2017). Due to differences in geography, economics, politics and many other aspects, social concerns and policy priorities vary enormously in different countries. In the global context, how countries set policy priorities in different stages of development, and how policy priorities align with the national development remain unknown. So, developing a global profiling for policy analysis can present the differences in core concerns of polices among countries, thus promoting further dialog and cooperation on the development of the international community in the future.

Bibliometric analysis has long been used as a statistical tool to systematically review scientific literature (Hood and Concepcion, 2001). A rigorous bibliometric analysis can provide systematic insights into previous publications, which can not only delve into the academic research community of active and influential researchers, but also identify the current research topics, and further explore potential directions for future research (Fahimnia et al., 2015). Bibliometrics has been widely applied in a wide range of sectors and specific domains, for example, mapping and visualizing the knowledge progress avenues and research collaboration patterns of cultural heritage (Vlase and Lähdesmäki, 2023), analyzing the sub-areas and core aspects of disease (Baskaran et al., 2021), visualizing and graphing the evolution of research related to sustainable development goals (Belmonte-Ureña et al., 2021), and studying policies, such as agricultural policy (Fusco, 2021), medical information policy (Yuxi et al., 2018), and science, technology and innovation policy (Zhang et al., 2016). However, the research trajectory and focus of policy analysis around the world remain a black box. In the present paper, a bibliometric analysis was performed from three dimensions: time, intensity, and scope, which referred to hot point changes over time, the quantity of research and the core concerns of policy, respectively.

In the present paper, a bibliometric analysis of a large number of studies on historical policy analysis was performed to answer the questions: (1) What core concerns are reflected in the policy analysis and how does these core concerns reflect real-world social problems? (2) How do these core concerns change over time? (3) What are the differences in core concerns among countries and what drives those differences? From an evolutionary perspective, this paper aims to uncover the past and present policy problems of concern and the relevant possible options, thus providing a clue for future policy analysis. The analysis of the evolution and differences in policy problems among countries may provide a view of the development context of different countries and put forward new inspiration and hope for further dialog and cooperation on the development of the international community in the future. Furthermore, another possible key sustainability implication with respect to the core concerns of policy analysis is to provide a reference for exploring the gaps between academic research and policy agenda.

## Methods

### Literature research

In the present study, Web of Science (WOS) Core Collection database was used for data retrieval (Vlase and Lähdesmäki, 2023). This research was conducted in four steps. Firstly, articles related to policy analysis were searched to select the most cited ones, which reflect the most influential research and the cutting-edge knowledge over time. MerigÓ et al. (2016) and Markard et al. (2012) weighted the most citation in an absolute term that means the total citations of all time. According to Fusco (2021) and Essential Science Indicators, the most citation was weighted in a relative term, which means the citation number in the publication year. The top 1% papers, compared to other articles in the academic field published in the same publication year, were included in this study following the refining principle of Essential Science Indicators, ensuring that the impact of these articles does not fade with time. Secondly, the selected papers were further screened, and narrowed down to different collected datasets for in-depth analysis according to the results of screening. Thirdly, statistical analysis and network visualization of authorship, organization and geographical distribution, topics and their chronological trends in each dataset were performed using VOSviewer software, which is freely available to construct and visualize bibliometric network (see www.vosviewer.com) (Van-Eck and Waltman, 2010). Lastly, the association between policy analysis and academic articles was explored in different fields.

### Dataset construction

Originally, a total of 118,535 articles related to policy analysis were retrieved using the strategy “TS = (policy analysis)”. For further discipline analysis, the most cited articles were selected with the quick filtering toolbar of WOS. Consequently, 1287 most cited papers of policy analysis were included in dataset 1. Then co-citation analysis of journals was performed to provide clues for discipline research (Supplementary Table 2). Accordingly, policy analysis-related articles from journals in the medicine field were selected for dataset 2, and 7963 articles were finally included. Similarly, 15,705 articles from journals in the field of environment were included in dataset 3; 6253 articles from journals in the field of energy in dataset 4; 1268 articles from journals in the field of economy in dataset 5; and 2243 articles from multidisciplinary journals in dataset 6. According to Journal Citation Reports of WOS, multidisciplinary journals refer to those journals in which articles involve at least two disciplines, such as *Ecological Economics* that involves ecology and economics. The search strategy of each database is shown in Table 1.

**Table 1:** Search strategies for the six datasets.

No.	Number of articles	Search strategies
#0	118,535	TS = (policy analysis)
#1	1287	Most cited papers of policy analysis
#2	7963	TS = (policy analysis) AND SO = (*The Lancet* OR *JAMA* OR *The Lancet Infectious Diseases* OR *PLOS One* OR *The Lancet Global Health* OR *The Lancet Public Health* OR *BMJ-British Medical Journal* OR *The Lancet Oncology* OR *Annals of Internal Medicine* OR A*merican Journal of Public Health* OR *Social Science Medicine* OR *Health Affairs* OR *JAMA Internal Medicine* OR *PLOS Medicine* OR *American Journal of Preventive Medicine* OR *The New England Journal of Medicine* OR *BMC Public Health* OR *Bulletin of the World Health Organization* OR *International Journal of Epidemiology* OR *Implementation Science*)
#3	15,705	TS = (policy analysis) AND SO = (*Journal of Cleaner Production* OR *Science of The Total Environment* OR *Global Environmental* OR *Global Environmental Change-human Human and Policy Dimensions* OR *Transportation Research Part D: Transport and Environment* OR *Environmental Modeling Software* OR *Atmospheric Chemistry and Physics* OR *Environmental Science and Pollution Research* OR *Earth System Science Data* OR *Remote Sensing of Environment* OR *Climatic Change* OR *Nature Climate Change* OR *Environmental Science Technology* OR *Journal of Environmental Management* OR *Ecological Indicators* OR *Ecology and Society* OR *Landscape and Urban Planning* OR *Sustainable Production and Consumption* OR *Sustainability* OR *Resources Conservation and Recycling*)
#4	6253	TS = (policy analysis) AND SO = (*Sustainable Cities and Society* OR *Energy Policy* OR *Applied Energy* OR *Renewable Energy* OR *Energy*)
#5	1268	TS = (policy analysis) AND SO = (*International Journal of Production Economics* OR *Transportation Research Part A: Policy and Practice*)
#6	2243	TS = (policy analysis) AND SO = (*Ecological Economics* OR *Nature* OR *PNAS* OR *Nature Communications* OR *European Journal of Operational Research*)

*TS* for Topic Search (including title, abstract, and keywords), *SO* for Publication Name.

### Network visualization

**Fig. 2:**
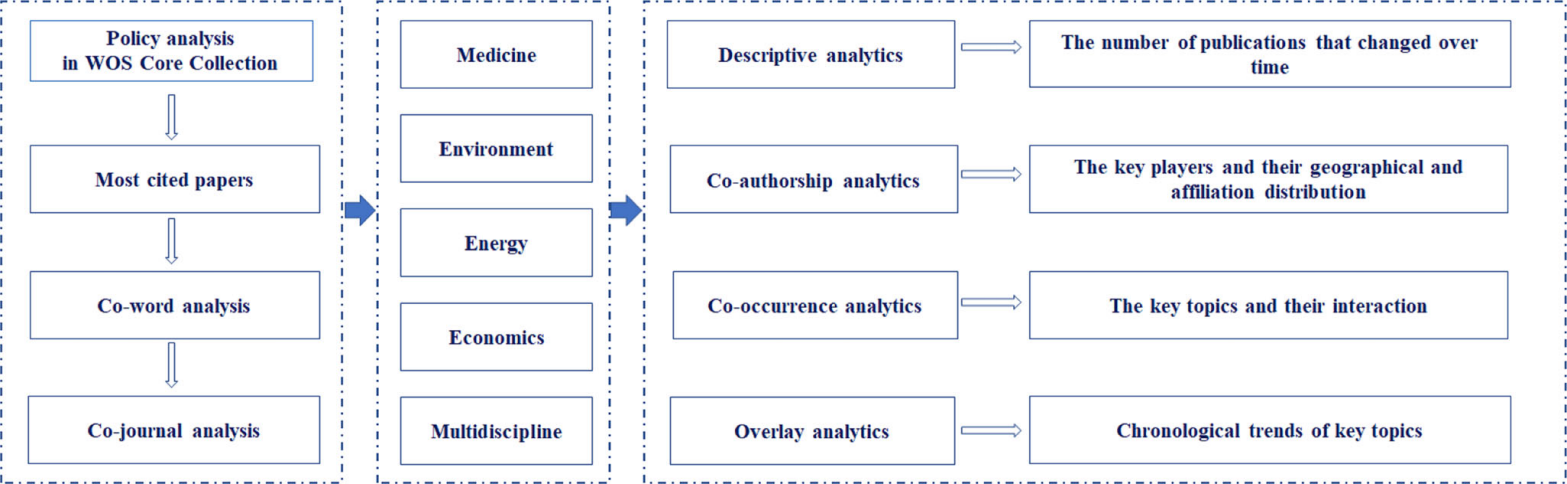
The research framework for multidisciplinary investigation in policy analysis.

Publication information of policy analysis was presented, including publication number, countries and organizations of key players, which reflects the value of and actual needs for policy analysis. Then, VOSviewer was used for network visualization of co-authorship, co-occurrence and citation. Co-authorship analysis for organizations and countries, which met the thresholds identified more than 5 articles for further investigation of the key players’ geographical distributions and their collaboration patterns. Co-occurrence analysis for all keywords based on the frequency of keywords used in the same article was carried out for topic mining (Kern et al., 2019). Citation analysis was performed to investigate the citation attributes received by other items. Meaningless or common terms were removed (Zhang and Porter, 2021). The research framework is shown in Fig. 2.

## Results

### Publication information of policy analysis

**Fig. 3:**
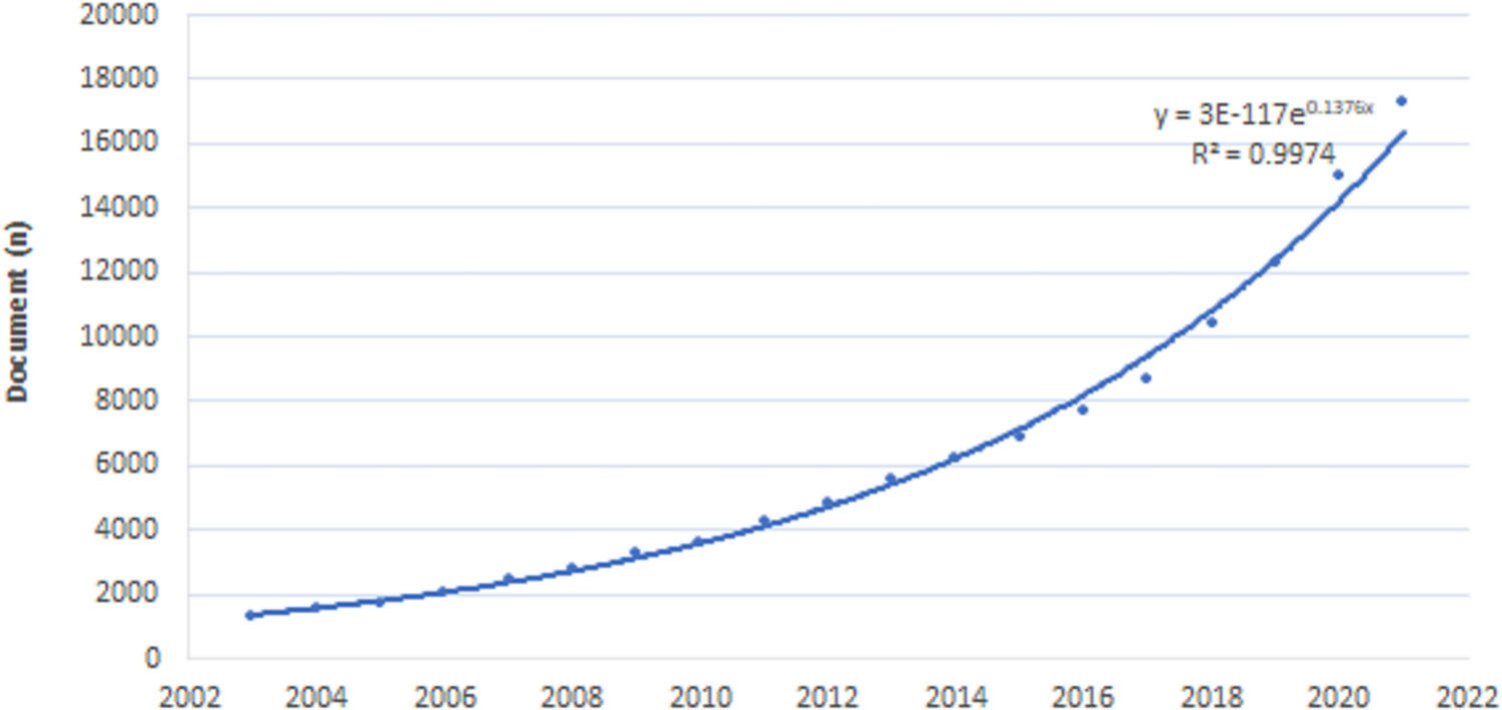
Trend of the number of publications on policy analysis between 2003 and 2021. Source : Data was collected from Web of Science (WOS) Core Collection database on the topic (TS) “policy analysis”.

Firstly, the publication number of policy analysis was determined. A total of 118,535 policy analysis articles were published between 2003 and 2021 (Fig. 3), showing a surge in the development of policy analysis with an exponential growth rate of 53.98 and 84.03% in the last 5 years (2017–2021) and 10 years (2012–2021), respectively.

**Fig. 4:**
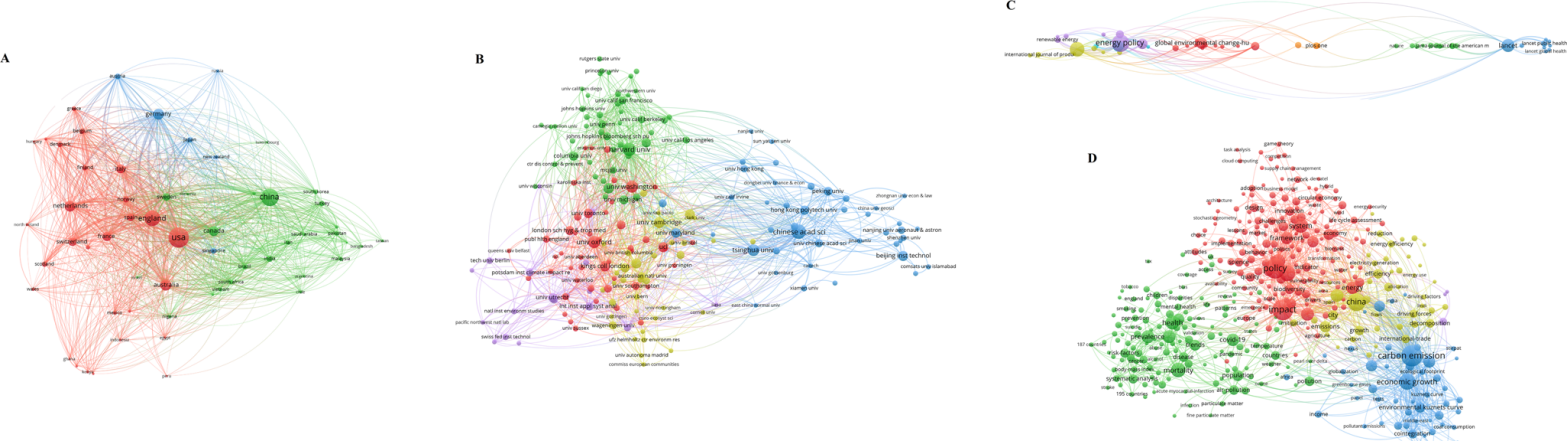
Profiling for policy analysis. **A** Co-authorship analysis for countries; **B** Co-authorship analysis for organizations; **C** Citation network; **D** Co-occurrence network.

For network construction, 1287 most cited papers were screened. The collaboration network of countries was visualized and illustrated, showing that 112 countries have published the most cited policy analysis articles. As for the co-authorship of countries and organizations, 2286 universities were identified, and 193 of them from 59 countries met the criteria of network analysis, among which the universities from the USA (University of Washington, Harvard University), the UK (University of Oxford, University of Cambridge) and China (University of Chinese Academy of Sciences) had the largest number of links and the strongest willingness to cooperate with other organizations (Fig. 4A, B and Supplementary Table 1). The willingness of cooperation not only meets the needs of academic research, but also conforms to the general expectations of the international community. Citation analysis for sources identified 51 journals from five different fields (Fig. 4C and Supplementary Table 2), in which environment-related journals accounted for the largest number (e.g., *Journal of Cleaner Production, Science of The Total Environment*, *Global Environmental Change-Human and Policy Dimensions*, *Transportation Research Part D: Transport and Environment* and *Environmental Modeling & Software)*, followed by medicine-related journals (*The Lancet*, *JAMA*, *The Lancet Infectious Diseases*, *PLOS One* and *The Lancet Global Health)*, the journals of energy science (*Sustainable Cities and Society*, *Energy Policy*, *Applied Energy*, *Renewable Energy* and *Energy*), the journals of economy (*International Journal of Production Economics* and *Transportation Research Part A: Policy and Practice*), and then several multidisciplinary journals (*Ecological Economics*, *Nature*, *PNAS, Nature Communications* and *European Journal of Operational Research*).

In the co-word network of policy analysis, four main clusters were displayed: the blue cluster concerned with environmental policy problems; the green cluster related to medicine (e.g., public health, prevalence and mortality of disease); the red cluster centering policy, such as policy framework, policy systems, and policy implementation; and the yellow cluster mainly concerned with energy (e.g., energy consumption, energy efficiency and electricity generation) (Fig. 4D and Table 2). Simultaneously, more details related to real-world social issues were also found, such as the common and core concerns about carbon emission, economic growth, prevalence and mortality of disease. Additionally, management is in the spotlight (e.g., system, framework, efficiency and challenge).

**Table 2:** Top 10 most frequent keywords in policy analysis.

No.	Label	Weight <total link strength>	Weight <occurrences>	Pub. Year
1	carbon emission	1082	152	2017
2	impact	750	145	2017
3	policy	708	144	2016
4	economic growth	685	88	2018
5	model	418	87	2016
6	system	402	74	2017
7	mortality	354	74	2016
8	management	349	66	2016
9	health	291	65	2016
10	prevalence	288	59	2016

### Publication information of policy analysis in different fields

**Fig. 5:**
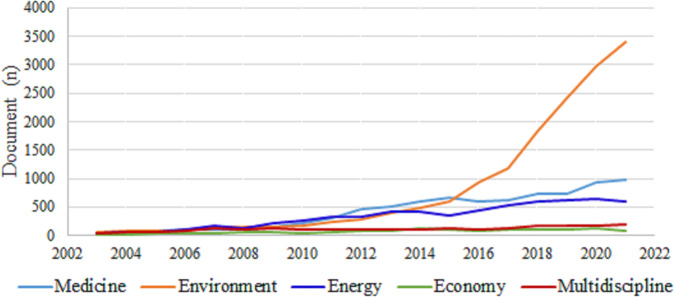
Publication dynamics of policy analysis-related articles in the fields of medicine, environment, energy, economy and multidiscipline between 2003 and 2021.

Policy analysis-related articles mainly involved the fields of medicine, environment, energy, economy and multidiscipline. The publication information in different fields was investigated. First, the volume growth trend over time was traced. Generally, a growing number of articles were published annually. The most obvious growth was found in policy analysis in environment, followed by medicine and energy, and the growth in economy and multidiscipline was relatively stable (Fig. 5). Specifically, the first increase in the publication number of policy analysis in medicine was seen in 2009, and then a steady growth was maintained, followed by a second acceleration after 2019, which may relate to the pandemic of H1N1 influenza and COVID-19, respectively (WHO, 2012; Wouters et al., 2021). A great growth in environmental policy analysis was observed after 2015, and a linear growth after 2017. In energy policy analysis, the first increase occurred in 2009, reaching a peak in 2013, followed by a second increase in 2016, reaching another peak in 2020. Then the publication information about organizations and countries was explored. The top five countries and institutions with the largest number of policy analysis articles in different fields are presented in Supplementary Table 3. The results showed that the USA, the UK and China attached great importance to policy analysis in all of these fields.

### Policy analysis in the field of medicine

**Fig. 6:**
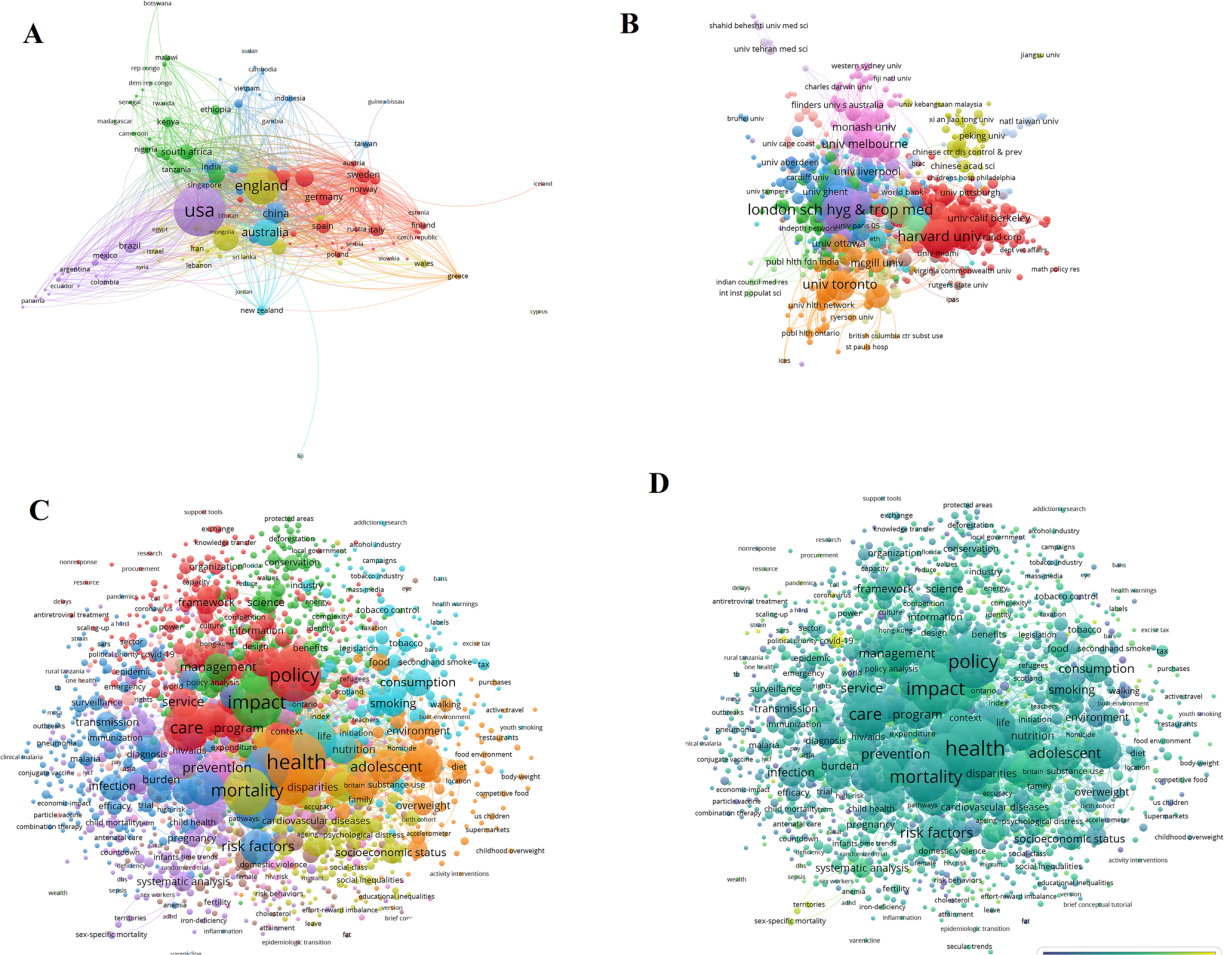
Profiling for health policy analysis. **A** Co-authorship analysis for countries; **B** Co-authorship analysis for organizations; **C** Co-occurrence network; **D** Overlay network.

A total of 8381 organizations from 177 countries contributed to medical policy analysis. Further investigation showed that universities from the UK (e.g., University of London, London School of Hygiene & Tropical Medicine and University College London), the USA (e.g., Harvard University and University of California San Francisco), Canada (e.g., University of Toronto) and Australia (e.g., University of Melbourne, University of Sydney) contributed the most to medical policy analysis with the greatest willingness to collaborate both domestically and internationally. By contrast, Chinese universities, such as Peking University, University of Chinese Academy of Sciences and Zhejiang University, were more prone to domestic collaboration (Fig. 6A, B).

Co-occurrence analysis of keywords showed that of the 16,719 keywords identified from 7963 retrieved items, 1778 keywords met the threshold. In addition to the three core topics “medicine”, “policy” and “health” (e.g. health policy, public health), the mortality, prevalence, risk factors as well as prevention of diseases have been the key focus of medical policies. Additionally, the issues of children and adolescents, such as physical activity, overweight and childhood obesity, have also attracted medical scientists and policy analysts. Figure 6D shows the average annual overlay network of keywords. The most recent concerns are the prevalence of COVID-19 and relevant topics associated with SARS-CoV-2 and coronavirus. Moreover, sex-specific mortality, life satisfaction and affordable care act are also the hot topics in recent years (Fig. 6C, D).

### Policy analysis in the field of environment

**Fig. 7:**
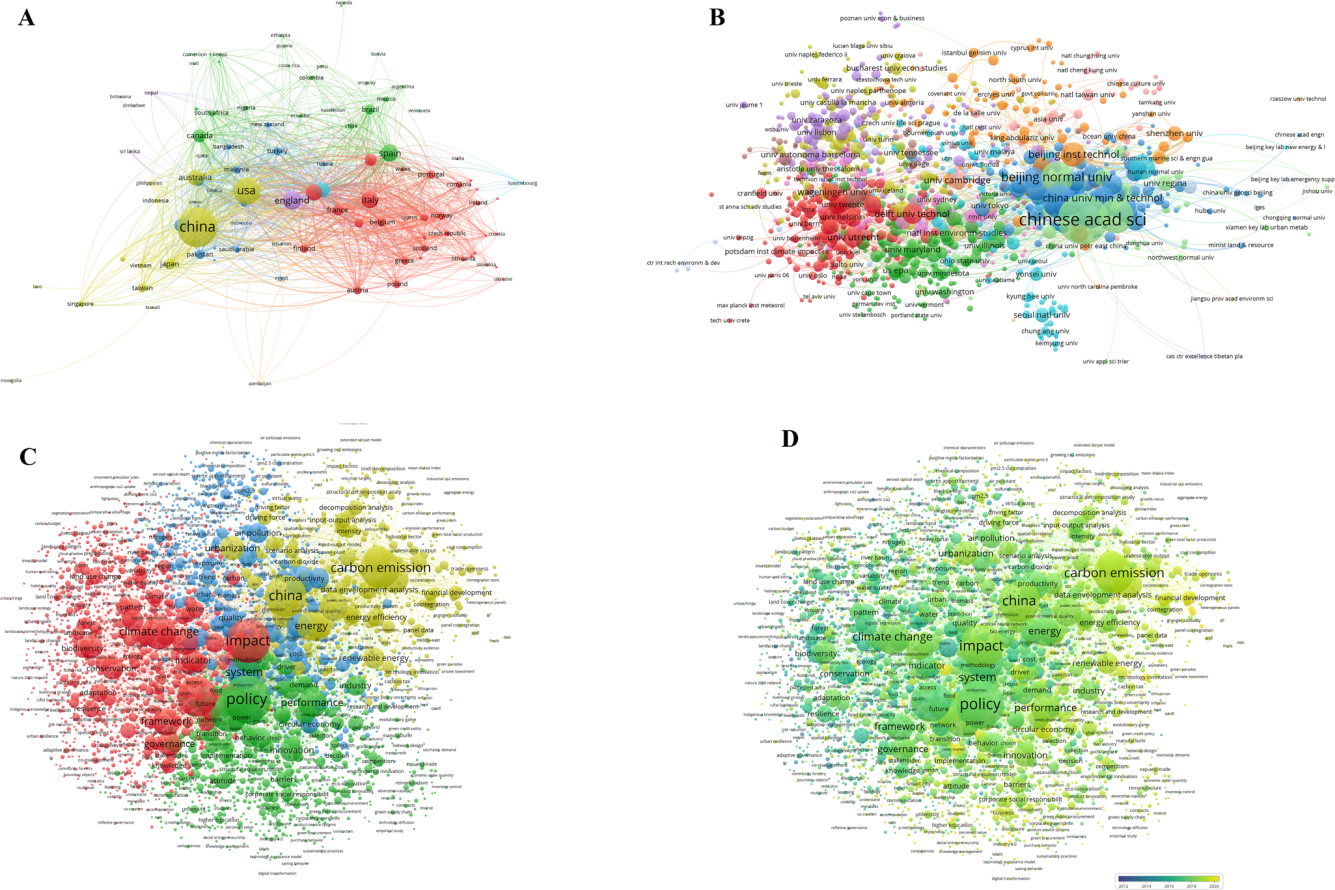
Profiling for environmental policy analysis. **A** Co-authorship analysis for countries; **B** Co-authorship analysis for organizations; **C** Co-occurrence network; **D** Overlay network.

Co-authorship analysis showed that 9060 organizations from 160 countries contributed to environmental policy analysis, among which universities from China played a key role, especially University of Chinese Academy of Sciences, Tsinghua University, Beijing Normal University, North China Electric Power University and Beijing Institute of Technology (Fig. 7A, B and Supplementary Table 3). Of the 44,213 keywords in retrieved 1 5705 articles related to environmental policy analysis, 3638 met the threshold of keyword co-occurrence analysis. The co-word network showed that apart from the words with vague meanings such as “policy”, “impact” and “management”, “carbon emission”, “climate change” and “sustainability” were the most visible in the network. Note that the terms like “energy”, “economic growth” and “urbanization” were also easy to notice (Fig. 7C). The analysis for the average annual overlay showed that “kyoto protocol”, “acid deposition” and “policy development”, etc. were earlier terms, while “plastic pollution”, “Cross-Sectionally Augmented Autoregressive Distributed Lag” and “population structure”, though lightly weighted, were the most recent ones. The color of overlay network visualization of environmental policy analysis appeared to be yellow, indicating that environmental problems have attracted researchers all over the world in past decades (Fig. 7D). The abovementioned results demonstrated the positive attitude of policy analysts and indicated a shift of their attention over time, possibly due to the evolution of environmental problems.

### Policy analysis in the field of energy

**Fig. 8:**
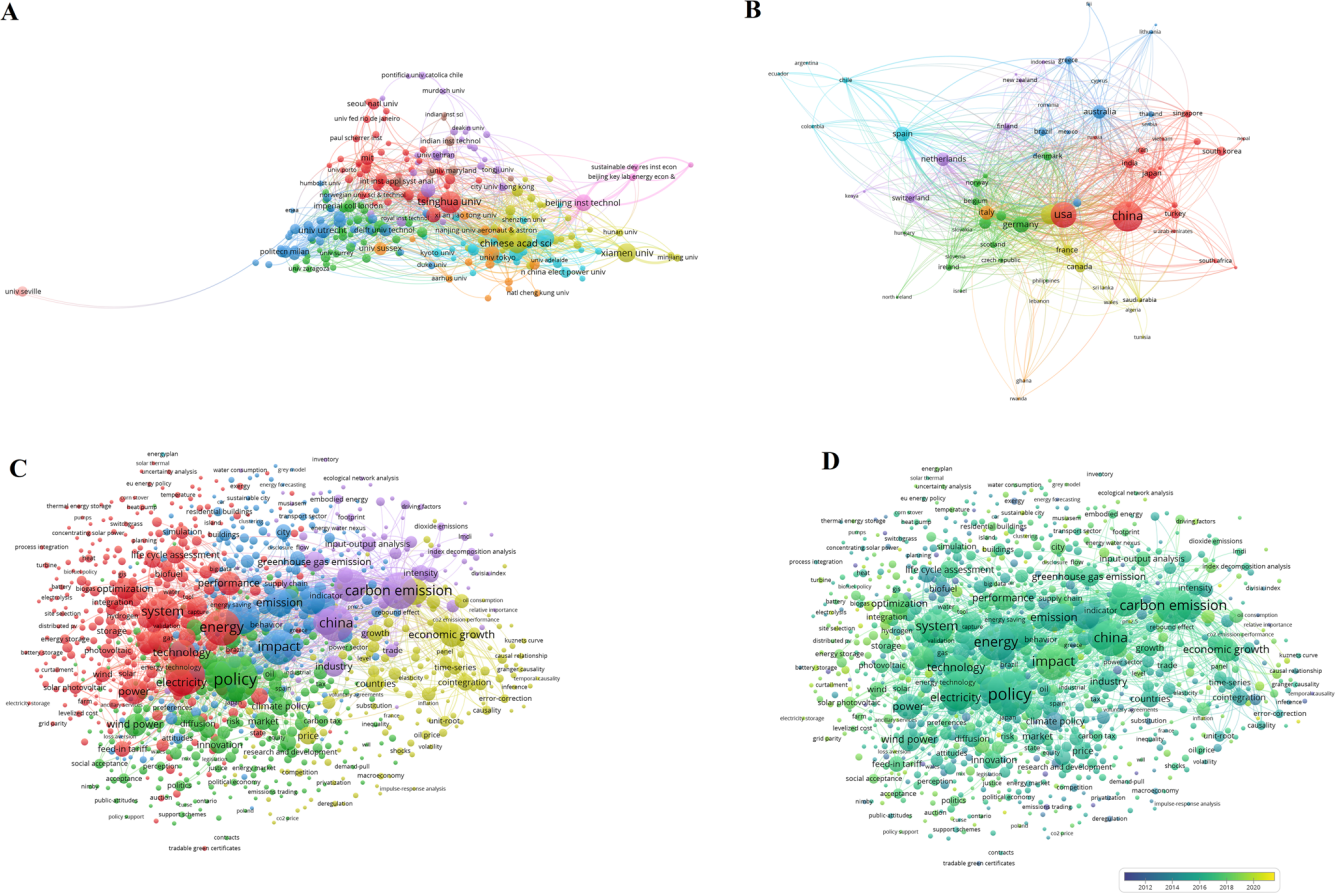
Profiling for energy policy analysis. **A** Co-authorship analysis for countries; **B** Co-authorship analysis for organizations; **C** Co-occurrence network; **D** Overlay network.

The collaboration network showed that 3668 organizations from 117 countries performed policy analysis in energy. The top five organizations were Tsinghua University, University of Chinese Academy of Sciences, Xiamen University, North China Electric Power University and Beijing Institute of Technology, all of which showed strong willingness to collaborate both domestically and internationally. The network showed that there was complex knowledge interaction and flow in the citation of energy policy analysis (Fig. 8A, B). Of the 15,027 keywords in retrieved 6253 articles, 1225 met the threshold. Co-occurrence network (Fig. 8C) revealed that policy analysis in energy was primarily focused on the demand for renewable energy (such as “wind power”, “solar power”, “bioenergy”) due to emission (e.g. “carbon emission”, “greenhouse gas emission”) and energy consumption. The terms “restructuring”, “discount rates” and “kyoto protocol” were early noticed by researchers, and the analysis of kyoto protocol was performed earlier in energy than that in ecology. Then, “green power”, “green certificates” and “energy policy analysis” gradually came into the eyes of analysts. Similarly, the prevalence of COVID-19 was the greatest concern of energy policy analysts, followed by “energy communities” and “renewable energy consumption” (Fig. 8D).

### Policy analysis in the field of economy

**Fig. 9:**
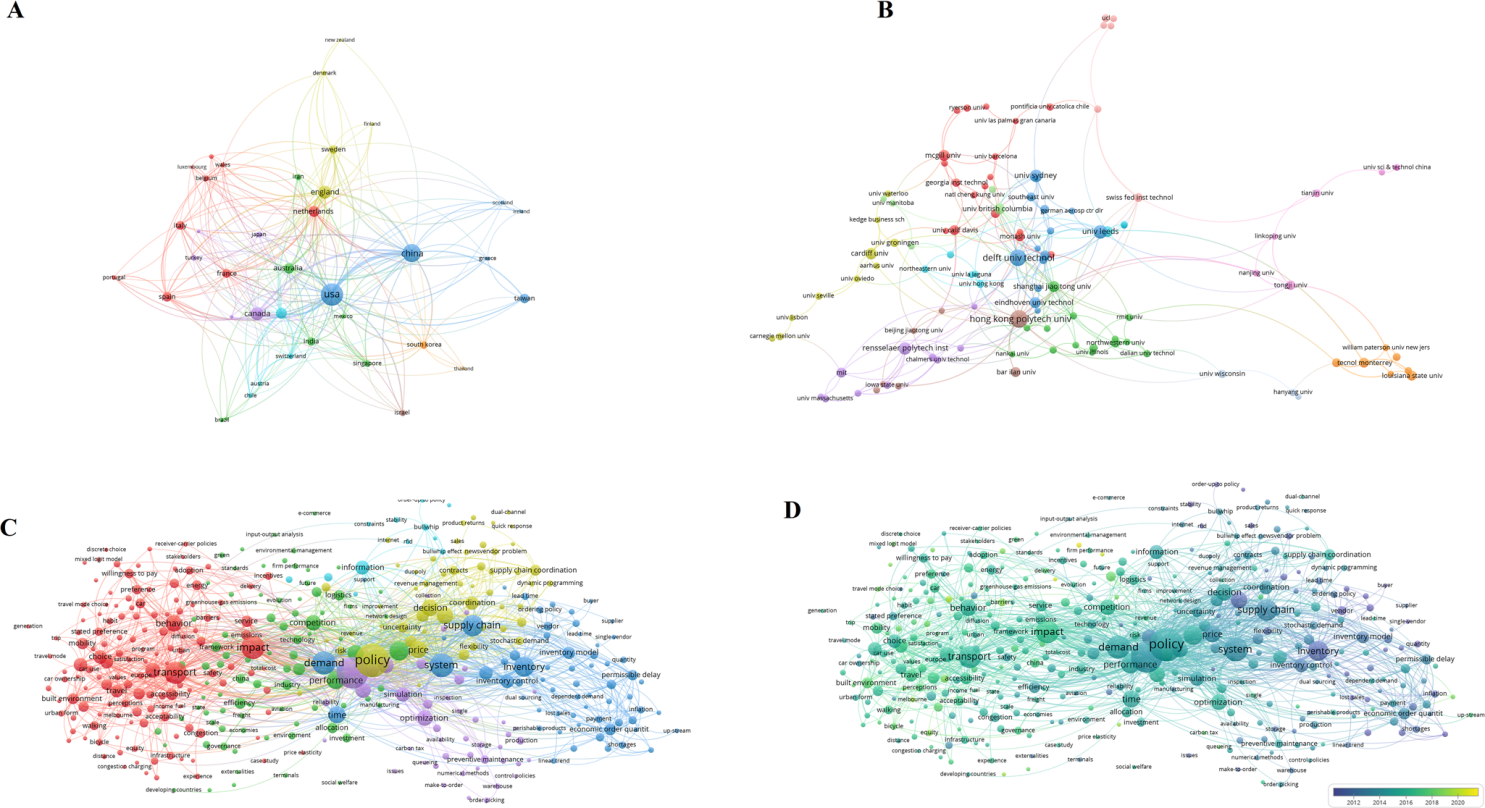
Profiling for economic policy analysis. **A** Co-authorship analysis for countries; **B** Co-authorship analysis for organizations; **C** Co-occurrence network; **D** Overlay network.

1144 organizations from 67 countries were found to contribute almost the same to policy analysis in economy. Hong Kong Polytechnic University, Delft University of Technology, University of Leeds, Rensselaer Polytechnic Institute and University of Sydney had the largest number of publications. Hong Kong Polytechnic University, Delft University of Technology, University of British Columbia, University of Sydney and Rensselaer Polytechnic Institute had the highest collaboration (Fig. 9A, B). Of the 5970 keywords in retrieved 1268 papers, 395 met the threshold. The co-word network showed that in addition to the general words frequently used in articles (e.g. “policy”, “impact”, “system”), the specific words reflecting the most common topics for policy problem of economy were “transport” (associated with vehicles, public transport, travel behavior, etc.), “supply chain” (related to supply chain management, supply chain coordination, green supply chain, etc.), and “inventory” (related to the model, control and system of inventory, etc.) (Fig. 9C). The overlay network analysis showed that economic policy analysts had an early interest in inventory-related topics and the issue of supply chain management, but has been concerned with the sustainability of supply chain management only in recent years. Additionally, topics like “circular economy”, “life-cycle assessment”, “industry 4.0” and “automated vehicles” also attracted scholars’ attention. (Fig. 9D).

### Policy analysis in multidiscipline

**Fig. 10:**
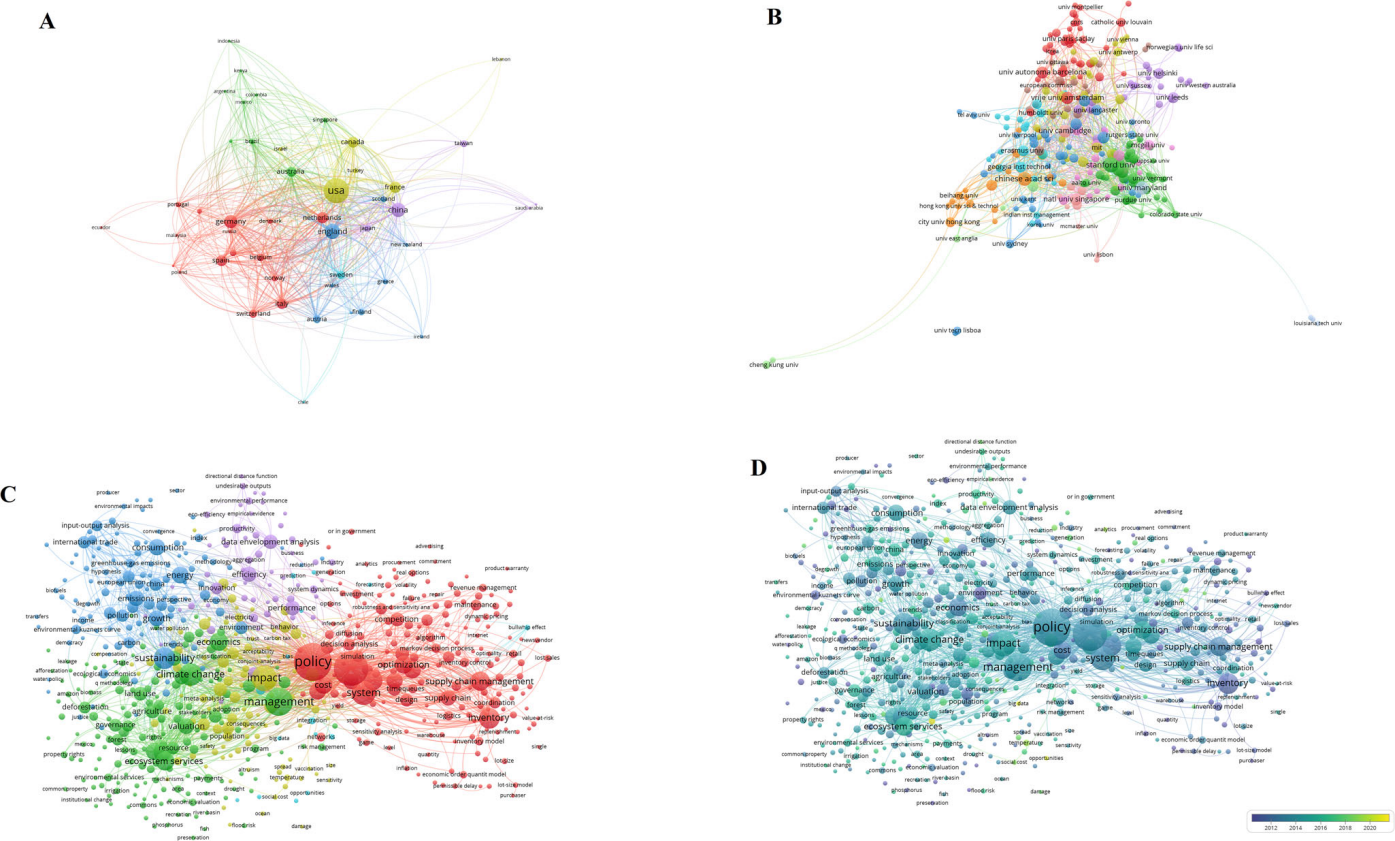
Profiling for multidisciplinary policy analysis. **A** Co-authorship analysis for countries; **B** Co-authorship analysis for organizations; **C** Co-occurrence network; **D** Overlay network.

In the co-authorship network, universities such as Stanford University, University of Chinese Academy of Sciences, University of Maryland, University of California, Berkeley and University of Cambridge had the most publications and a high collaboration. University of California Irvine had fewer publications but relatively higher link, showing that this university was strongly willing to cooperate with other organizations (Fig. 10A, B). Of the 9467 keywords in retrieved 2243 articles, 648 met the threshold. This multidisciplinary research revealed the relationship between economy, environment and energy. However, there were obstacles to extend the relationship between them. Co-word network demonstrated that the policy analysis articles published on the multidisciplinary journals were mainly focused on the topics of “climate change”, “sustainability” and “inventory”. The term “climate change” is mainly related to issues of environmental resources (e.g., land use, deforestation, biodiversity), greenhouse gas emission (especially carbon emission) and energy consumption. The term “sustainability” is mainly connected with the relationship between environmental resources and economic growth. In addition to COVID-19, the terms “big data” and “circular economics” were on the cut edge (Fig. 10C, D).

## Discussion

Policy analysis aims to understand what is the governments’ focal point, investigate why and how governments issue policies, evaluate the effects of certain policies (Browne et al., 2019), and reflect political agenda driven by social concerns or international trends (Kennedy et al., 2019). In this study, a bibliometric analysis of a large number of publications on historical policy analysis was carried out to explore the policy problems of concern and the relevant possible options from an evolutionary perspective, and provide a guide for future research. From 2003 to 2021, the number of publications on policy analysis grew exponentially. Before 2011, little attention was paid to policy analysis, but in recent decades, more importance has been attached to policy analysis around the world due to increasingly prominent social problems, especially the human health needs, degradation of environment, energy consumption and the relationship between economy, energy and environment.

From the perspective of global visibility, the policy analysis in medicine has received increasing attention from scholars from 8381 organizations of 177 countries, indicating that health problems, though not numerically dominant, have the widest coverage. Among these countries, the USA, the UK, Australia, Canada and China are the major contributors. The developed countries, such as the USA, the UK, Canada and Australia, have strongly supported addressing complex public health issues by developing effective policy responses (Moore et al., 2011; Atkinson et al., 2015). Typically, they spend the most on health, with 12318, 5387, 5905 and 5627 dollars per capital, respectively, while the developing countries spend relatively less, such as 894 dollars per capital in China and 231 dollars per capital in India (OECD, 2022). Great attempts have been made to analyze the burden of prevalence and mortality of diseases such as cancer, cardiovascular diseases and diabetes both globally and regionally (Yusuf et al., 2020; Rudd et al., 2020; Kearney et al., 2005). Other health issues of women, children and adolescents have been monitored and measured for years in many countries that respond to the Countdown to 2030 (Countdown to 2030 Collaboration, 2018). In addition, the worldwide outbreak of epidemics such as H1N1 influenza and COVID-19 pandemic has caused excess mortality and enormous social and economic costs all over the world, which greatly affect social policy and reveal the fragility of health systems to shocks (Wouters et al., 2021; Chu et al., 2020). By analyzing the global burden of disease, scholars have recommended policy-makers to give priority to the prevention and management of relevant diseases (Kearney et al., 2005).

Environmental policy analysis involving 15,705 articles has attracted largest attention from policy analysts and scientists. Greenhouse gas emission (mainly carbon emission) resulting in climate change and environmental degradation remains to be the most threatening and urgent issue, and has attracted attention of governments and the society (Tang et al., 2021; Ahmad et al., 2019). Different countries issued different climate policies aiming to reduce greenhouse gas emissions. The Kyoto protocol, ratified by 180 countries, committed to reduce the GHG emissions by 5% by 2012, compared with the 1990 emission levels (Kuosmanen et al., 2009). In the EU climate policy framework in 2014, the carbon emissions were projected to reduce by 40% by 2030, and by 80% by 2050 (European Council, 2014). The relationship between urbanization and environmental pressure was observed in the present research. During urbanization, the consumption of resources such as land, water and fuel has increased significantly, causing serious ecological pressure such as climate change, loss of biodiversity, land erosion and pollution. With the acceleration of economic growth and social commercialization, urbanization further increases the demands for housing, food, transportation, electricity and so on, which in turn aggravates the ecological pressure because of natural resource consumption, climate change, over-extraction and pollution (Ahmed et al., 2019; Wang et al., 2019). Hence, urbanization policies with restrictions on unplanned urban sprawl are under the way (Ahmed et al., 2020).

Energy is another big agenda for policy analysis. The close connection between energy and emission has been presented noticeably in this study. Governments have come to a consensus that there should be greater balance between ecological purity, energy supply and economic well-being if a country strives for healthy and sustainable economic development (Alola and Joshua, 2021). New environmental policies should be designed to control environmental pollution through reducing pollutant emissions and sustaining economic growth, and should be incorporated into governments’ macro policies (Halicioglu, 2009). Transformation of energy sector was on agenda to meet the ambitious goals (Cong, 2013). The UK, the USA and China are the global leaders in reducing actual emissions and increasing energy supply. In the USA, the shale revolution brought global attention to energy supply and remains to be a driving force for energy policies. Low-cost shale gas combined with the policy support for renewables have notably reduced CO_2_ emissions over the past decades. Environmental deregulation is another central focus, which may affect the trajectory of greenhouse gas emission (International Energy Agency, IEA, 2019a, 2019b). In the UK, the policy objectives of actual emission reduction, carbon budgets setting and investment in energy technology and innovation reflect the ambition for decarbonization (IEA, 2019a, 2019b). As is known, China’s GDP grows rapidly, which has multiplied more than 170 times since the founding of the People’s Republic of China 73 years ago. However, the extensive economic growth mode depending on the primary and secondary industries has put high pressure on environment, such as large amounts of consumption and pollution (He et al., 2016; Yue et al., 2021; Yu and Liu, 2020). Data showed that the greenhouse gas emission (OECD, 2020) and air pollution exposure (OECD, 2022) in China have been far higher than those in other countries for a long time, posing great challenges to both the government and scholars. A specific policy package, such as the “Atmosphere Ten Articles”, “Soil Ten Plan” and “Water Ten Plan” from 2013 to 2016, and the “Regulation on the Implementation of the Environmental Protection Tax Law of the People’s Republic of China” in 2017, has been issued by Chinese government, aiming to improve the ecological environment. Furthermore, goals for renewable energy production were also set by scholars. Jacobson suggested that wind, water and sunlight energy should be produced by 2030, and then replace the existing energy by 2050 (Jacobson and Delucchi, 2011), while Lund proposed that renewable energy (the combination of biomass with wind, wave and solar) should account for 50% by 2030, and 100% by 2050 (Lund and Mathiesen, 2009). However, it remains unclear how many countries can achieve their stated goals. Numerous studies have shown the efforts of governments and scholars to transform the resource and energy usage-driven economic expansion to sustainable development.

From the economics perspective, the environmental Kuznets curve (EKC) hypothesis demonstrates the relationship between environmental quality and economic output, which has been proved by empirical studies (Fodha and Zaghdoud, 2010; Saboori et al., 2012). Additionally, the relationship between economic growth and energy consumption has also been confirmed (Shahbaz et al., 2015). In recent years, countries have been facing the challenge of economic structural transformation. The mode of economic growth that relies on the consumption of natural resource and waste disposal seems increasingly outdated (McDowall et al., 2017). Circular economy, a new mode for reconciling environmental and economic imperatives, has come into the public eye and appears to meet the common vision of sustainable development. With the increase of requirements of sustainable development and circular economy, greening of supply chain management also faces challenges, including inventory management, mode of transportation, life-cycle assessment and coordination with other areas (Ghosh and Shah, 2012; Ghosh and Shah, 2015). Thus, providing support for green supply chain supplier deserves the attention from policy-makers and practitioners.

### Key findings

(1) Policy analysis has been a great concern of scholars for many years and has attracted increasing attention year by year, which reflects the value of and actual needs for policy analysis. (2) The world is facing common problems, which requires attention and efforts of the whole world, and a more harmonious social development such as the management of epidemics and complex disease, environmental-friendly development, green energy production and transformation from resource and energy usage-driven economic expansion to sustainable development is on the way. (3) Global profiling for policy analysis demonstrates that the central policy problems align with national development, which inspires further dialog and cooperation on the development of the international community in the future.

## Limitations

This study has limitations. First, keywords cannot fully reflect the essential intent of an article although they are the key points of a study. Therefore, using keywords as an element for bibliometric analysis is far from enough. Second, this paper deals with academic research of policy analysis, but whether it is fully consistent with the policy agenda is unexplored. Moreover, we have shown the correlations between different phenomena, but the underlying mechanism remains indefinable.

## Supplementary information

Supplementary information

## Data Availability

The datasets analyzed during the current study are available in the Dataverse repository (10.7910/DVN/XZMVMN).
