# Supplementary material for: What are the core concerns of policy analysis? A multidisciplinary investigation based on in-depth bibliometric analysis
Source: Humanit Soc Sci Commun. 2023 May 1;10(1):190. doi: 10.1057/s41599-023-01703-0 (PMC10150689; doi:10.1057/s41599-023-01703-0)
Supplement: Supplementary file 1 — Supplementary information [file 41599_2023_1703_MOESM1_ESM.docx]

Supplementary Table 1. Top 10 countries and organizations involved in policy analysis.

| **No.** | **Countries** | **Document (n)** | **No.** | **Countries** | **Document (n)** |
| --- | --- | --- | --- | --- | --- |
| 1 | The USA | 33816 | 6 | Germany | 6057 |
| 2 | China | 18670 | 7 | Netherlands | 5804 |
| 3 | The UK | 14757 | 8 | Italy | 5784 |
| 4 | Australia | 8797 | 9 | Spain | 5105 |
| 5 | Canada | 8394 | 10 | France | 4204 |
| **No.** | **Affiliations** | **Document (n)** | **No.** | **Affiliations** | **Document (n)** |
| 1 | University of London | 5470 | 6 | University of London, London School of Hygiene &Tropical Medicine | 1453 |
| 2 | University of California | 3445 | 7 | Johns Hopkins University | 1373 |
| 3 | Harvard University | 2531 | 8 | University of Oxford | 1355 |
| 4 | University of Chinese Academy of Sciences | 2269 | 9 | University of Sydney | 1332 |
| 5 | University of Toronto | 1787 | 10 | University of North Carolina | 1330 |

| Supplementary Table 2: The co-citation analysis of journals | | | | |
| --- | --- | --- | --- | --- |
| **Journals in the field of medicine** | **Journals in the field of environment** | **Journals in the field of energy** | **Journals in the field of economy** | **Journals of** **multidisciplinary science** |
| The Lancet | Journal of Cleaner Production | Sustainable Cities and Society | International Journal of Production Economics | Ecological Economics |
| JAMA | Science of The Total Environment | Energy Policy | Transportation Research Part A: Policy and Practice | Nature |
| The Lancet Infectious Diseases | Global Environmental Change-Human and Policy Dimensions | Applied Energy |  | PNAS |
| PLOS One | Transportation Research Part D: Transport and Environment | Renewable Energy |  | Nature Communications |
| The Lancet Global Health | Environmental Modelling & Software | Energy |  | European Journal of Operational Research |
| The Lancet Public Health | Atmospheric Chemistry and Physics |  |  |  |
| British Medical Journal | Environmental Science and Pollution Research |  |  |  |
| The Lancet Oncology | Earth System Science Data |  |  |  |
| Annals of Internal Medicine | Remote Sensing of Environment |  |  |  |
| American Journal of Public Health | Climatic Change |  |  |  |
| Social Science & Medicine | Nature Climate Change |  |  |  |
| Health Affairs | Environmental Science & Technology |  |  |  |
| JAMA Internal medicine | Journal of Environmental Management |  |  |  |
| PLOS Medicine | Ecological Indicators |  |  |  |
| American Journal of Preventive Medicine | Ecology & Society |  |  |  |
| The New England Journal of Medicine | Landscape and Urban Planning |  |  |  |
| BMC Public Health | Sustainable Production and Consumption |  |  |  |
| Bulletin of the World Health Organization | Sustainability |  |  |  |
| International Journal of Epidemiology | Resources Conservation and Recycling |  |  |  |
| Implementation Science |  |  |  |  |

| Supplementary Table 3: Top 5 countries and organizations with the largest number of published articles in different fields. | | | | | |
| --- | --- | --- | --- | --- | --- |
|  | **Medicine** | **Environment** | **Energy** | **Economy** | **Multidiscipline** |
| **Countries** | The USA (3329) | China (5526) | China (1461) | The USA (335) | The USA (754) |
|  | The UK (1680) | The USA (2446) | The USA (1146) | China (224) | The UK (286) |
|  | Australia (897) | The UK (1248) | The UK (656) | The UK (119) | China (252) |
|  | Canada (838) | Italy (1086) | Germany (443) | Canada (113) | Germany (224) |
|  | China (628) | Spain (1017) | Spain (343) | Germany (84) | Netherlands (163) |
| **Affiliations** | University of London, London School of Hygiene & Tropical Medicine (312) | Chinese Academy of Sciences (728) | Tsinghua University (130) | Hong Kong Polytechnic University (38) | Stanford University (37) |
|  | Harvard University (266) | Tsinghua University (310) | Chinese Academy of Sciences (103) | Delft University of Technology (34) | University of Maryland (27) |
|  | University of California, San Francisco (212) | Beijing Normal University (288) | Xiamen University (93) | University of Leeds (23) | Chinese Academy of Sciences (30) |
|  | University Toronto (193) | North China Electric Power University (193) | North China Electric Power University (83) | Rensselaer Polytech Institute (20) | University of California, Berkeley (26) |
|  | University College London (183) | Beijing Institute of Technology (169) | Beijing Institute of Technology (74) | University of Sydney (19) | University of Cambridge (26) |
